# Prevalence and factors associated with menstruation-related school absenteeism among adolescent girls in rural northern Ghana

**DOI:** 10.1186/s12905-021-01418-x

**Published:** 2021-08-02

**Authors:** Maxwell Tii Kumbeni, Florence Assibi Ziba, Joana Apenkwa, Easmon Otupiri

**Affiliations:** 1 Ghana Health Service, Nabdam District Health Directorate, Nangodi, Ghana; 2 School of Nursing, University for Development Studies, Tamale, Ghana; 3 School of Public Health, Kwame Nkrumah University of Science and Technology, Kumasi, Ghana

**Keywords:** Adolescent girls, Menstruation, School absenteeism, Prevalence, Factors, Ghana

## Abstract

**Background:**

Although menstruation is a normal physiological process that begins in girls during adolescence, it has the potential to negatively impact on the self-esteem and education of girls particularly those from low- and middle-income countries. We investigated the prevalence and factors associated with menstruation-related school absenteeism among adolescent girls in the Talensi district of rural northern Ghana.

**Methods:**

We conducted a cross-sectional survey among 705 adolescent girls aged 12–19 years who had attained menarche. The sample size was estimated using Epi Info version 6 at 95% confidence interval and a 5% margin of error. A two-stage sampling technique was employed to recruit participants. We conducted univariate and multivariate logistic regression models to determine factors associated with menstruation-related school absenteeism which was defined as “being absent from school due to menstruation-related issues during the last menstruation.”

**Results:**

The prevalence of menstruation-related school absenteeism was 27.5%. School absenteeism ranged from one to seven days during the menstrual period. Older adolescent girls, (aOR = 2.38, 95% CI 1.29–4.40), use of cloth as a sanitary material at the last menstruation, (aOR = 3.21, 95% CI 2.22–4.63), and cultural restriction, (aOR = 2.54, 95% CI 1.76–3.67) were associated with higher odds of menstruation-related school absenteeism. Meanwhile, girls from moderate income parent(s), [aOR = 0.57 95% CI 0.34–0.94] had lower odds of menstruation-related school absenteeism. Mother’s education and privacy in school were only significant at the univariate level.

**Conclusions:**

The prevalence of menstruation-related school absenteeism highlights the need for interventions aimed at improving the availability of sanitary pads for girls, eliminating cultural restrictions associated with menstruation, and also improving parent(s) income level.

## Introduction

The population of adolescents represent 23% of the 1.1 billion people living in sub-Saharan Africa, and about a half of them are girls between the ages of 10–19 years [1]. Adolescence is an important milestone which occurs with psychological and biological changes that require prompt sexual and reproductive health (SRH) attention [2]. However, many adolescent girls in low- and middle-income countries (LMICs) face numerous challenges with regards to SRH services [3, 4]. Although there are global efforts to improve educational opportunities for adolescent girls, one of the challenges these girls in LMICs face is managing their menstruation amid other pubertal changes [5–7].

Several studies have documented the impact of menstruation on the education of adolescent girls, with challenges of menstrual hygiene management affecting girls’ school attendance. For instance, a recent study in Uganda by Miiro and colleagues [8], suggested that the prevalence of school absenteeism among girls during menstruation was 59%. These girls missed school from one to seven days in a month during their menstrual period. According to Mohammed and others, the prevalence of menstruation-related school absenteeism in Ghana was  40% [9]. Montgomery and colleagues [10], also reported that school girls stayed away from school for up five days in a month during their menstrual period in Ghana. Other studies have reported similar findings in most LMICs [5, 7, 11].

In the school setting, lack of clean, private, gender specific, water, sanitation and hygiene (WASH) facilities are documented to be associated with menstruation-related school absenteeism among girls [10, 12–14]. Lack of sanitary pads has also been widely reported to be a major contributory factor to school absenteeism among girls in their menstrual period [7, 10, 14, 15]. Other documented factors such as cultural restrictions, age of girls and mother’s education are said to play an influence on menstruation-related school absenteeism [7, 16]. Self-reported factors such as menstrual pain, fear of staining clothing and fear of being teased at school have also been reported to be associated with school absenteeism [8, 9, 17].

There are limited studies in Ghana on menstrual issues among school going adolescent girls. More so, these studies have focused on dysmenorrhea, menstrual knowledge and menstrual hygiene management [16–18]. The limited studies that have reported on menstruation-related school absenteeism in Ghana have only assessed sanitary pad with menstrual education as the factors associated with  school absenteeism [9, 10]. An assessment and understanding of the full range of these factors  is an important step towards developing specific interventions to curb the rather high burden of menstruation-related school absenteeism. The aim of our study was to investigate the prevalence and factors associated with menstruation-related school absenteeism among adolescent girls in a rural setting of northern Ghana. The findings will be relevant towards improving menstrual health and education of adolescent girls particular in poor and rural settings.

## Materials and methods

### Study design and population

A cross-sectional study was conducted in junior high schools in the Talensi district of Ghana, from July to September, 2016. A total of 705 school girls aged 12–19 years who had attained at least their first menstruation participated in the study.

### Study setting

Talensi district is in Upper East Region of Ghana with Tongo as the district capital. It has a total population of 86,182 out of which women of reproductive age is 20,684. The total adolescent population in the district stands at 19,160 out of which 10,942 are between 10–14 years and 8,668 are between 15–19 years. The female adolescent population is 8,927 for which 4,978 are within 10–14 years while 3949 are between 15–19 years. The district has four health centers, one polyclinic, 16 Community-based Health Planning and Services (CHPS) compounds and one district hospital. Health is also accessed from places like medicine stores and Traditional healers.

There are two Senior High Schools, 37 Junior High, 48 Primary and 49 Kindergarten schools. In 2015, the total girl child population, aged 12-19 years, in Junior High schools was 2,239. The district has seven market squares and menstrual sanitary materials are sold in all the markets. The commonly sold sanitary material is the sanitary pad. The district also has a total of 228 functional toilet facilities which comprises of 14 public toilets, 157 KVIPs, 57 institutional toilets and 64 water closets. There are a total of 33 sanitation sites in the district and each site has a waste disposal container sited at a suitable area for collection of solid domestic waste from various points in the communities.

### Sample size and data collection

Epi Info Version 6 was used to calculate the sample size at a prevalence of 50%, using 95% confidence interval and a 5% margin of error. The estimated sample was 730 considering a design effect of two and a 10% non-response rate. A two-stage sampling strategy was employed to select participants. At stage one, a sample frame comprising of 37 eligible schools was obtained from the Ghana Education Service in the district out of which 15 schools were systematically selected. The first school was randomly selected after which every third school was included in the study for up 15 schools. Registers for the selected schools were obtained and eligible participants teased out of them. A simple random sampling technique was used to select the individual study participants on the day of data collection. The sampling was done with proportionate allocation of size to the study populations in the schools. Selected participants responded to self-administered questionnaire at the premises of their various schools.

### Outcome variable

The outcome variable of interest was menstruation-related school absenteeism. This was defined as “being absent from school due to menstruation-related issues during the last menstruation.” Our outcome variable was categorized as “yes” for school absenteeism and “no” for no school absenteeism.

### Predictor variables

The predictor variables were age, religion, mother’s education, parent(s) income status, type of sanitary material used during the last menstruation, privacy in school and cultural restrictions. Age was categorized as, 12–15, 16–17, 18–19; religion (Christianity, Islam, Traditional), and mother’s education (no formal education, primary education, secondary or higher education). Parent(s) income status was categorized into low (none of the parents are engaged in any form of job apart from subsistence farming), moderate (one or both parents are engaged in petty trading within the locality in addition to subsistence farming) and high (one or both parents are engaged in medium or high business, or formal sector jobs such as teaching, nursing, practicing medicine). The type of sanitary material used during the last menstruation (sanitary pad, cloth). Privacy in school was defined as “toilet facility or room with roof and door locks which is accessible to only females” and was categorized as “yes” and “no”. Cultural restriction was also defined as any one or more of the following restrictions during menstruation “prevented from going to school, prevented from visiting religious grounds, prevented from playing with colleagues and prevented from eating certain types of foods”. “Yes” was assigned to restrictions during menstruation and “no” to not being restricted. The variable selection was informed by previous studies [10, 12, 14, 15].

### Data analysis

Data were analyzed using STATA Version 15 (College Station, TX: Statacorp LLC). Univariate and multivariate analyses were done to assess the relationships between the predictors and our outcome variable. Statistical significance was considered at a p-value of < 0.05.

## Results

### Characteristics of the study sample

Majority (53.3%) of the girls were aged 16–17 years, and 92.9% of them were Christians. Most of the girls’ mothers had no formal education (74.8%), and majority (73.5%) of the girls came from low-income status parent(s). About two-thirds (65.5%) of the girls used sanitary pads during their last menstruation, 65.7% said they had privacy in school during menstruation and 43.5% indicated to have faced cultural restrictions during their menstrual periods. The prevalence of school absenteeism by girls due to menstruation was 27.5%. The number of days absent from school in the last menstruation were; one to two days (49.0%), three to four days (37.1%) and five to seven days (13.9%) [Table 1].

**Table 1:** Characteristics of the study sample (n = 705)

Variables	Frequency (%)
Socio-demographic characteristics	
Age	
12–15	258 (36.6)
16–17	376 (53.3)
18–19	71 (10.1)
Religion	
Christianity	655 (92.9)
Islamic	35 (5.0)
Traditional	15 (2.1)
Mother’s education	
No formal education	527 (74.8)
Primary education	154 (21.8)
Secondary or higher education	24 (3.4)
Parent(s) income status	
Low	518 (73.5)
Moderate	153 (21.7)
High	34 (4.8)
Menstruation-related characteristics	
Type of sanitary material used during the last menstruation	
Sanitary pad	464 (65.8)
Cloth	241 (34.2)
Privacy in school	
No	242 (34.3)
Yes	463 (65.7)
Cultural restrictions	
No	398 (56.5)
Yes	307 (43.5)
Menstruation-related School absenteeism	
No	511 (72.5)
Yes	194 (27.5)
Number of days absent in a month (n = 194)	
1–2 days	95 (49.0)
3–4 days	72 (37.1)
5–7 days	27 (13.9)

### Factors associated with menstruation-related school absenteeism

Girls aged 18–19 years were 2.38 times more likely to have stayed away from school during their menstrual period compared to those aged 12–15 years, (aOR = 2.38, 95% CI 1.29–4.40). The girls with parents of moderate-income status had 43% lower odds of staying away from school during their menstrual period when compared with those from low-income status, (aOR = 0.57, 95% CI 0.34–0.94). Additionally, the girls who used cloth as sanitary material in their last menstruation were 3.21 times more likely to have been absent from school during their menstrual period compared with those who used sanitary pad, (aOR = 3.21, 95% CI 2.22–4.63). The girls who encountered cultural restrictions during their menstrual period were significantly more likely to be absent from school when compared with those who did not encounter any cultural restrictions, (aOR = 2.54 95% CI 1.76–3.67). Mother’s education and privacy in school were significant at univariate level but lost significance in the multivariate model (Table 2).

**Table 2:** Factors associated with menstruation-related school absenteeism (n = 705)

Variables	Unadjusted or (95% CI)	Adjusted or (95% CI)
Age		
12–15	1.00	1.00
16–17	1.52 (1.05–2.21)	1.49 (0.99–1.23)
18–19	2.54 (1.44–4.50)	2.38 (1.29–4.40)*
Religion		
Christianity	1.00	1.00
Islamic	1.40 (0.68–2.69)	0.99 (0.45–2.17)
Traditional	1.35 (0.45–4.00)	1.40 (0.43–4.49)
Mother’s education		
No formal education	1.00	1.00
Primary education	0.57 (0.37–0.89)	0.71 (0.44–1.15)
Secondary or higher education	0.32 (0.09–1.11)	0.59 (0.15–2.27)
Parent(s) income status		
Low	1.00	1.00
Moderate	0.43 (0.27–0.69)	0.57 (0.34–0.94)*
High	0.13 (0.03–0.56)	0.31 (0.07–1.40)
Type of sanitary material used during the last menstruation		
Sanitary pad	1.00	1.00
Cloth	3.68 (2.56–5.28)	3.21 (2.22–4.63)*
Privacy in school		
No	1.00	1.00
Yes	0.68 (0.48–0.96)	0.70 (0.48–1.02)
Cultural restrictions		
No	1.00	1.00
Yes	2.56 (1.81–3.68)	2.54 (1.76–3.67)*

* = significance at P-value < 0.05; 1 = Reference category

## Discussion

Our study investigated the prevalence and factors associated with menstruation-related school absenteeism among adolescent girls. The prevalence of menstruation-related school absenteeism was at least one-in-four girls, and the days spent away from school during their last menstruation ranged from one to seven days. Older adolescent girls (18–19 years), use of cloth as a sanitary material in the last menstruation and cultural restrictions were associated with higher odds of menstruation-related school absenteeism, while parent(s) income status (moderate-income) was associated with lower odds of menstruation-related school absenteeism. Mother’s education and privacy in school were not associated with menstruation-related school absenteeism at the multivariate regression level.

Our findings of more than a quarter of the adolescent girls missing out school days during their menstrual period highlights the many challenges girls face in managing their menstruation alongside schooling. Missing out school days means missing out critical lessons in class and sometimes examinations, which may never be recovered after they have returned to school. This puts them at a further disadvantage to the boys and has the potential to stall the academic performance of these girls [7, 15]. More so, consistent monthly menstruation-related school absenteeism may lead to class repetition and subsequent school dropout of these girls due to poor academic performance [15]. The prevalence of menstruation-related school absenteeism in our study is lower than the 40% as found by Mohammed and colleagues [9], in Ghana, 40% in India by Vashisht and colleagues [7] and 54% in Ethiopia by Tegegne and Sisay [15]. However, our finding is a little higher when compared to the 20% menstruation-related school absenteeism as reported in Uganda [8]. The discrepancy could be due to differences in income levels, cultural settings, school infrastructure and time variations of the studies.

We also found that older adolescent girls were more likely to be absent from school due to menstruation-related issues when compared with the younger ones. Miiro and colleagues [8], in their study in Uganda reported that missing school during girl’s menstrual period was more common among the older adolescents. In a similar study in Indonesia by Davis and colleagues [5], it was also found that girls in higher grades were more likely to report school absenteeism due to menstruation during their last menstrual period. In older adolescents, there is an increased tendency to function independently of their parents and so they tend to make such critical decisions regarding sexual and reproductive health without the involvement of their parents [19]. Older adolescents may therefore shy away from discussing and seeking support from their parents regarding menstrual-related challenges they might be facing in school even if the parents can provide support such as sanitary pad among others. This might be a possible explanation why older adolescent girls are more likely to stay at home during their menstrual period compared to the younger ones. Increased parental income was associated with reduced school absenteeism during menstruation. Girls from parent(s) with moderate-income status are likely to get funds that will cater for their monthly sanitary pads, and this might enhance their school attendance. Our findings aligns with a similar study in Northern Ethiopia [15].

The use of cloth in the last menstruation was associated with higher odds of menstruation-related school absenteeism among the girls. Hennegan and colleagues [6], suggested that the use of unclean cloth as a menstrual absorbent was associated with fear of odour, inability to concentrate in school and low confidence in answering questions in class, and this may contribute to abstaining from school during menstruation. In a similar study, Montgomery and colleagues [20], reported that school attendance during menstruation was higher among girls who were supplied with sanitary pads compared to those who were not supplied with sanitary pads. These findings are not surprising because most girls feel the sanitary pad is superior to cloth and therefore they are more comfortable using sanitary pad while in school without the fear of staining themselves and being teased [21]. Similar findings are reported elsewhere in Ghana [10], Ethiopia [15], India [22], and Bhutan [23].

The prevalence of menstruation-related school absenteeism in our study was more common among girls who were culturally restricted during menstruation. The period of adolescence is of particular concern because in most LMICs, cultural restrictions, misconceptions, myths and taboos create barriers which prevents girls from understanding the scientific facts about menstruation. These restrictions limit their daily activities and have the potential to negatively affect their self-esteem, reproductive health and school attendance [24, 25]. For example, in certain cultural settings, menstruating girls are excluded from religious functions, water bodies, certain foods, family homes and from using sanitation facilities meant for public. It is also a taboo for girls to look in the mirror or bath during their menstrual period [24–27]. These girls will not be comfortable to go to school if they have not bathed particularly for a good number of days due to fear that they may be smelly and be teased at school. Studies in certain parts of India have also reported that menstruating girls are prevented from going to school until the period of menstruation is over [26, 27].

Our study had some limitations. The main limitation of the study is that it was a cross-sectional study, and we cannot establish causal relationships. Also, our study did not consider some aspects of WASH such as water and soap availability at school. Furthermore, our variables were self-reported and recall bias might have been introduced. However, we expect recall bias to impact less on our outcome variable as it was measured in relation to the last menstrual period.

## Conclusion

More than a quarter of the girls stayed out of school during their menstrual periods. The number of menstruation-related school absenteeism during the last menstruation varied from one to seven days. Menstruation-related School absenteeism was more prevalent among girls who used cloth as a menstrual material in their last menstruation, the older adolescents and those who faced cultural restrictions during their menstrual periods. However, menstruation-related school absenteeism was less common among those whose parent(s) had moderate-income. At policy level, interventions should be aimed at improving the availability of sanitary pads for girls, eliminating cultural restrictions associated with menstruation, and also improving parent(s) income level. We recommend future research on the impact of menstruation-related school absenteeism on the academic performance of school girls.

## Data Availability

Data is available from the corresponding author upon request.

## Abbreviations

LMIC: Low-and-middle income countries
SHR: Sexual and reproductive health
